# A Comparative Study on the Microscale and Macroscale Mechanical Properties of Dental Resin Composites

**DOI:** 10.3390/polym15051129

**Published:** 2023-02-23

**Authors:** Shuogeng Yan, Kun Wang, Zhengzhi Wang

**Affiliations:** 1 Department of Engineering Mechanics, School of Civil Engineering, Wuhan University, Wuhan 430072, China; 2 Wuhan University Shenzhen Research Institute, Shenzhen 518108, China

**Keywords:** photopolymerization, degree of conversion, nanoindentation, mechanical properties, gradient boundary layer, mechanical reinforcement

## Abstract

Dental resin composites are universal restorative materials, and various kinds of fillers are used to reinforce their mechanical properties. However, a combined study on the microscale and macroscale mechanical properties of dental resin composites is missing, and the reinforcing mechanism of the composites is still not fully clarified. In this work, the effects of the nano-silica particle on the mechanical properties of dental resin composites were studied by combined dynamic nanoindentation tests and macroscale tensile tests. The reinforcing mechanism of the composites was explored by combining near-infrared spectroscopy, scanning electron microscope, and atomic force microscope characterizations. It was found that the tensile modulus increased from 2.47 GPa to 3.17 GPa, and the ultimate tensile strength increased from 36.22 MPa to 51.75 MPa, with the particle contents increasing from 0% to 10%. From the nanoindentation tests, the storage modulus and hardness of the composites increased by 36.27% and 40.90%, respectively. The storage modulus and hardness were also found to increase by 44.11% and 46.46% when the testing frequency increased from 1 Hz to 210 Hz. Moreover, based on a modulus mapping technique, we found a boundary layer in which the modulus gradually decreased from the edge of the nanoparticle to the resin matrix. Finite element modeling was adopted to illustrate the role of this gradient boundary layer in alleviating the shear stress concentration on the filler–matrix interface. The present study validates mechanical reinforcement and provides a potential new insight for understanding the reinforcing mechanism of dental resin composites.

## 1. Introduction

Dental restorative materials have experienced the development from silver amalgam and ceramic materials to new polymer composite materials [1,2,3]. Among them, light-cured resin composites have become the current mainstream dental restoration materials because of their outstanding characteristics, such as superior esthetics, low cost, environmental-friendly characteristics, simple repair operation, and good biocompatibility [4,5,6,7,8]. Pure resin materials are disadvantageous in their mechanical properties, such as strength, stiffness, durability, and wear resistance. Many reinforcing strategies have, therefore, been developed for the resin materials. One of the most common strategies is to add various inorganic fillers, such as silica glass, quartz, ceramic, metal, pre-polymerized particles, and/or natural minerals in diverse shapes and sizes, into the resin matrix [9,10,11,12,13,14,15,16].

The mechanical properties of dental resins reinforced by various fillers have been extensively studied in the literature [14,15,17,18,19,20,21,22]. For example, Zhu et al. [17] measured the flexural strength, flexural modulus, and compressive strength of the dental resin composites containing wrinkled mesoporous silica fabricated by a self-assembly technique. Chen et al. [18] evaluated the microhardness and the flexural strength to investigate the influences of N-acetyl cysteine on the mechanical properties of Poly-methylmethacrylate dental resins by a universal testing machine. Chang et al. [19] obtained the flexural strength and flexural modulus of Ca-doped mesoporous SiO_2_ dental resin composites by three-point bending tests and reported the compressive strength values. Liu et al. [20] explored the effects of different polyhedral oligomeric silsesquioxane additions on the mechanical properties of nano-silica reinforced dental composites based on nanoindentation and nanoscratch methods. They all found that the addition of various fillers can significantly enhance the mechanical properties of dental resin composites. However, there are still challenges when testing the mechanical properties via either the microscale or the macroscale methods alone. At the microscale, indentation size effects have a major influence on testing results; that is, different sizes of indenters may lead to different results. In some cases, the reinforcing fillers used may be larger than the indenter tip. In this situation, the results obtained may reflect only the mechanical properties of the fillers, not the composite material. At the macroscale, a large quantity of materials is required to prepare the samples. Moreover, the testing duration for macroscale tests is relatively long; for example, the load holding time for a typical triaxial compression test is usually several days [23]. Although plentiful research on the mechanical properties of dental resin composites has been reported [17,18,19,20,23,24,25,26,27,28], a systematic study that combines both macroscale and microscale tests is missing, and the connection between the microscale and macroscale performances is not clear. Therefore, the present study aims at combining the microscale and macroscale tests for a better understanding of the mechanical properties at different length scales and also the reinforcing mechanism of the dental resin composites. The hypothesis for the research is that nano-silica particles could enhance the mechanical properties of the resin composites, and there is an interactive relationship between the particles and matrix.

In this work, we explore the effect of silica nanoparticles on the mechanical properties of dental resin composites by both nanoindentation and macroscale tensile tests. The incorporation of silica nanoparticles can significantly enhance the stiffness, hardness, and strength of the dental composites even at low filler contents (up to 10 wt.% tested) [29,30,31], but the enhancement mechanism is unclear. Moreover, near-infrared (NIR) spectroscopy, scanning electron microscope (SEM), and atomic force microscope (AFM) techniques are adopted to characterize the polymerization kinetics, the morphology of fractured sections, and the modulus distribution of the particle–matrix interfaces, respectively. Finally, a possible strengthening mechanism for the resin composites based on the interactions between the nanoreinforcements and the resin matrix is proposed.

## 2. Materials and Methods

### 2.1. Materials

In this study, the resin matrix consisted of 70 wt.% Bisphenol A glycerolate di-methacrylate (Bis-GMA) and 30 wt.% Triethylene glycol dimethacrylate (TEGDMA) (both from BOC Sciences Inc., Shirley County, NY, USA). The photoinitiator camphorquinone (CQ) (0.2 wt.%) and the amine reducing agent ethyl 4-dimethylamino-benzoate (EDAB) (0.8 wt.%) (both from Sigma Aldrich, Milwaukee, WI, USA) were mixed with the matrix by a centrifugal mixer (DAC 150FVZ, FlackTek Inc., Landrum, SC, USA) for 20 min at 2500 RPM to obtain the uniform resin composites [32]. Then, the nano-SiO_2_ particles were respectively incorporated into the matrix with 0, 2, 4, 6, 8, and 10 wt.%, and the average diameter of the nanoparticles was 20 nm. The nano SiO_2_ particles with a mass ratio of 1% were added to the resin at a time; then, the mixtures were stirred evenly by the centrifugal mixer at 2500 RPM. Nanoparticles were then gradually added for centrifugal mixing again. The total amount of samples was about 8 g each time. All the mixtures were stirred for about 30 min by using the high-speed centrifuge to ensure the nanoparticles were dispersed homogenously in the resin matrix.

### 2.2. Preparation of Samples

**Figure 1:**
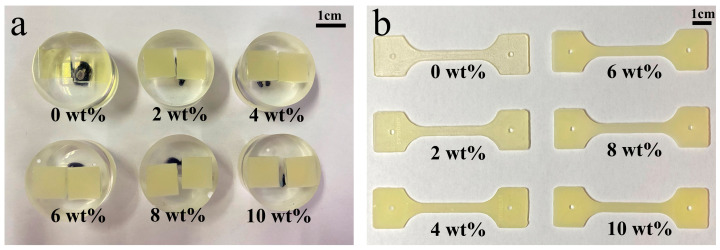
Photography of the samples adopted for (**a**) the nanoindentation tests and (**b**) the macroscale tensile tests.

For nanoindentation tests, the composites were poured into a square mold (10 mm × 10 mm × 5 mm) and irradiated for 60 s under the visible blue light with a light intensity of 50 mW/cm^2^. The cured samples were placed in a circular mold with a diameter of 25 mm, then sanded with silicon carbide papers (particle sizes of 800, 1200, 2500, and 5000 μm grit in sequence) and polished with diamond suspensions (particle sizes of 3, 1, and 0.3 μm). Then the surface smoothness of the sample was determined by a 50× optical microscope. The samples used in tensile tests were made from standard specimen mold and also irradiated for 60 s under the visible blue light with a light intensity of 50 mW/cm^2^. The sample sizes were confirmed by the standard tensile specimen proportions. The samples were all sanded until smooth to ensure the accuracy of the test results. The final samples tested by nanoindentation tests and macroscale tensile tests are shown in Figure 1a,b.

### 2.3. Experimental Methods

#### 2.3.1. Microscale Mechanical Properties Test

The microscale mechanical properties of the composites were investigated by nanoindentation tests. The nanoindentation tests were performed by the commercially available Triboindenter system (Ti-950, Hysitron, Minneapolis, MI, USA) with a diamond equilateral triangular indenter (Berkovich) and were carried out in frequency sweep force-control mode, in which the applied force was controlled in a pre-programmed manner, and the displacement continuously monitored; more complex dynamic load functions were created from it. The load function used in these tests was a fixed force of 3000 μN as well as a fixed dynamic load amplitude of 25 μN with a frequency from 1 Hz to 210 Hz. The force was divided into two parts, one was increased from 1 Hz to 10 Hz with the interval of 1 Hz, and another was increased with an increment of 20 Hz from 10 Hz to 210 Hz. Every data of modulus and hardness were derived from an average of 16 indentation points at four different locations on the samples. Four samples of each particle content were prepared for testing, and the error bars in the graphs were the standard deviation of all data. The distance between the indentation points was set to 40 μm to eliminate the interaction of adjacent indentations.

#### 2.3.2. Macroscale Mechanical Properties Test

The tensile tests were determined using the material universal testing machine (INSTRON 5969). The load was set as a force that increased with a constant rate of 2 MPa/s from 1 MPa to 20 MPa, the stress of the composites was calculated by force used in the test, and the cross-section size (5 mm × 1.5 mm) of the material and the strain were measured by extensometer. The tensile modulus could be obtained by the stress–strain curves. Moreover, the tensile strength of samples was obtained by the material universal testing machine with the same load rate. All experiments were performed 5 times; the experimental results were calculated by the average of the data, and the error bars in all graphs were obtained by calculating the standard deviation of all data.

#### 2.3.3. Degree of Conversion

**Figure 2:**
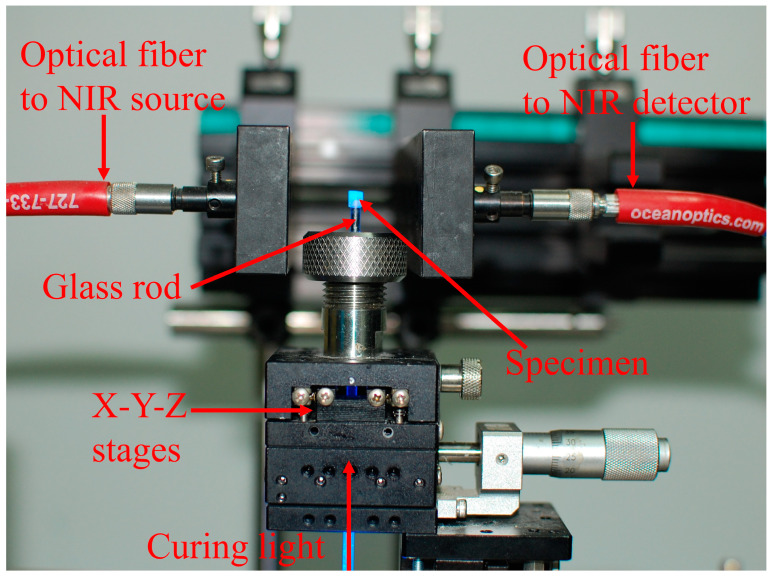
Photography of the in situ NIR spectroscopy for polymerization kinetics monitoring.

The degree of conversion (DC) of light-cured dental resin composites was determined by characterizing the NIR spectroscopy, and the NIR spectroscopy was measured by the NIR detection system, which consisted of an infrared (IR) light source (HL-2000, Ocean Optics, Dunedin, FL, USA) and a NIR detector (NIR Quest512-2.2, Ocean Optics, Dunedin, FL, USA). The diagram of the in situ NIR spectroscopy is shown in Figure 2. The composites were injected into a non-viscous Teflon tube (the height is 2 mm and the inner diameter is 2.5 mm), and the curing light was transmitted through the glass rod to the specimen. Two optical fiber cables were configured onto the sides of the Teflon tube; one was connected to the NIR spectrometer, and another was linked to the IR light. All samples were cured for 100 s under visible blue light with a light intensity of 50 mW/cm^2^.

The real-time DC was calculated according to the real-time change of absorption peak intensity of functional groups involved in the reaction. In this article, the area under the absorption peak of the methacrylic acid functional group, whose central position is 6165 cm^−1^, was used. The DC was calculated from the peak area before irradiation (Areapolymer) and the peak area at each time point in the process of photopolymerization (Areamonomer):

(1) $$\mathrm{DC}(\%)=(1-\frac{Area_{polymer}}{Area_{monomer}})\times 100$$

#### 2.3.4. Fracture Morphology of Dental Resin Composites

Fracture surfaces of the dental resin composites after tensile tests were evaluated with SEM (SIGMA500, Zeiss, Jena, Germany). Each sample was gold sputter-coated before observations to enhance the electrical conductivity of the samples.

#### 2.3.5. The Interaction between Particles and Matrix

PeakForce QNM pattern in AFM (Dimension Icon) was used to determine how particles strengthened the mechanical properties of composites. PeakForce QNM was used to obtain the modulus diagram of a particle-containing part of the matrix. It is a ground-breaking atomic force microscope imaging mode, and it is based on Bruker’s new proprietary Peak Force Tapping technology; the forces applied to the sample are precisely controlled, and a variety of probes can be used. This allows indentations to be limited to several nanometers in most cases, which both maintains resolution and prevents sample damage. Furthermore, a finite element simulation was also performed by ABAQUS to explain the strengthening mechanism of resin composites by particles.

## 3. Results

### 3.1. Mechanical Properties

#### 3.1.1. Microscale Mechanical Properties

**Figure 3:**
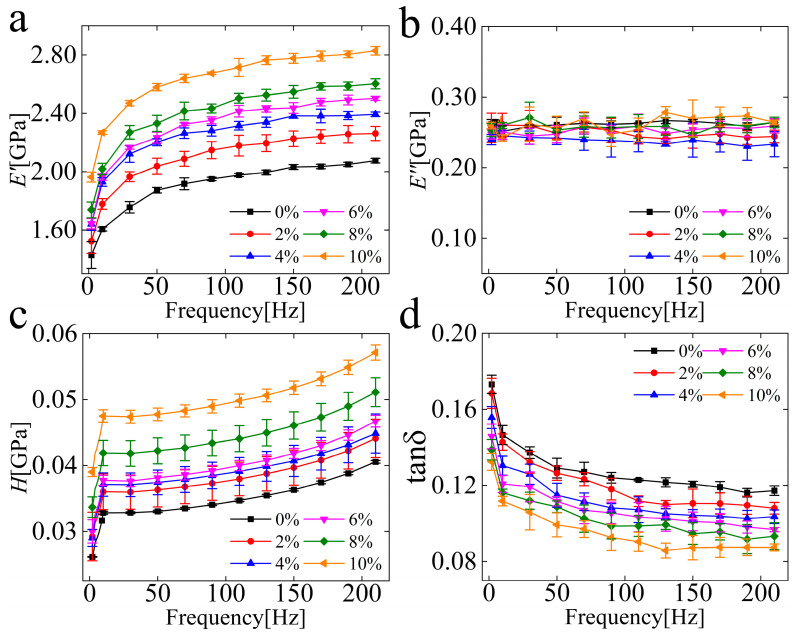
Results of dynamic nanoindentation tests. (**a**) Storage modulus; (**b**) Loss modulus; (**c**) Surface hardness; and (**d**) Loss factor of the composites with different nanoparticle contents as functions of the testing frequency.

The storage modulus (*E′*), loss modulus (*E″*), hardness (*H*), and loss factor tanδ curves of the dental resin composites with different particle contents versus the loading frequency graphs are all shown in Figure 3. The microscale mechanical properties increase with the filler contents. The storage modulus and hardness of the composites increased by 36.27% and 40.90%, with the particle contents added from 0% to 10%, respectively. The *E′* and H were also found to increase by 44.11% and 46.46% when the testing frequency increased from 1 Hz to 210 Hz. As shown in Figure 3b, the loss modulus does not change with the frequency, and the loss factor tanδ decreases with the frequency shown in Figure 3d; it could be attributed to the better particle/matrix adhesion.

#### 3.1.2. Macroscale Mechanical Properties

**Figure 4:**
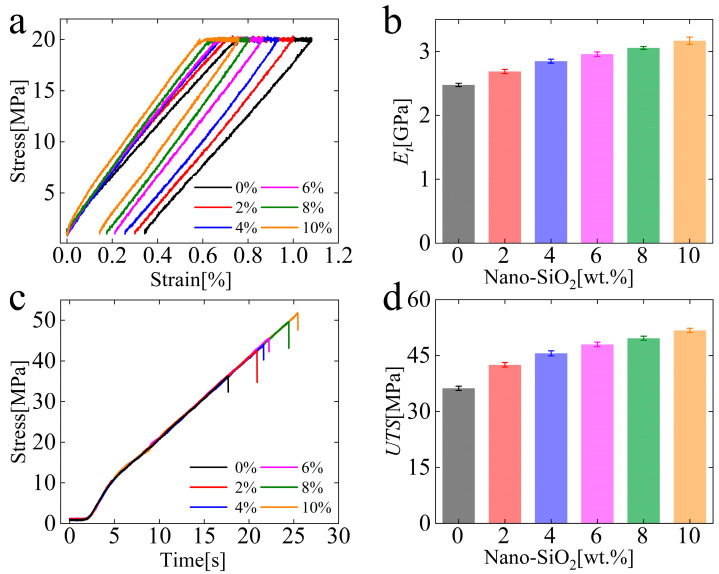
Results of the macroscale tensile test. (**a**) Stress-strain curves; (**b**) Stress-time curves; (**c**) Tensile modulus (Et); and (**d**) Ultimate tensile strength (UTS) of the composites with different nanoparticle contents.

The stress–strain curves, stress–time curves, and the macroscale mechanical properties of the composites are shown in Figure 4. From Figure 4a, it can be seen that the loading and unloading segments of the figure show a higher slope at higher particle content under the same loading method. During the unloading process, the samples undergo elastic recovery and present a decreasing irreversible deformation as the concentration of nanoparticle contents increases, which implies that the composites reinforced with nanoparticles exhibit much stronger deformation resistance than the pure one. Figure 4b shows the tensile modulus (*E_t_*) curves with different particle contents. Figure 4c shows that the unfilled sample breaks first, and the sample filled with 10% particles is the last one to break with the same loading rate. The ultimate tensile strength (*UTS*) curves with different particle contents are shown in Figure 4d. From Figure 4b, d, it could be found that the mechanical properties of the composites gradually increase with the addition of the particles. The tensile modulus and the ultimate tensile strength increase from 2.47 GPa and 36.22 MPa to 3.17 GPa and 51.75 MPa, with the particle content increasing from 0% to 10%, respectively. It can be seen that the addition of nano-SiO_2_ particles can enhance the macroscale mechanical properties of dental resin composites from Figure 4.

### 3.2. Strengthening Mechanism

#### 3.2.1. Degree of Conversion

**Figure 5:**
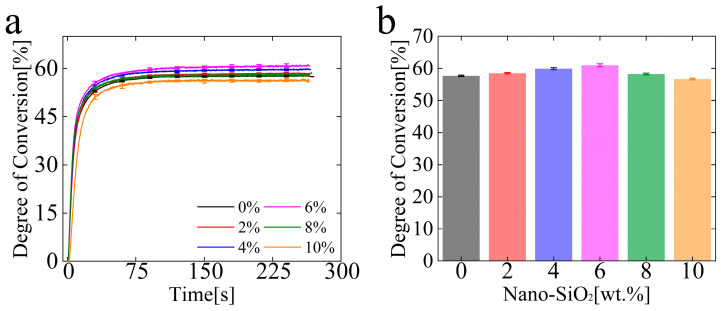
Results of the double-bond conversion. (**a**) Real-time development of the degree of conversion and (**b**) the final conversion of the composites with different nanoparticle content.

Figure 5 shows the degree of conversion of the composites. The real-time variation for DC with different particle contents is shown in Figure 5a. The DC increases with curing time and tardily levels off after approximately 80 s. The final stable value of DC with different particle contents is shown in Figure 5b. The final DC of the composites first increases from 57.58% to 60.97%, with an increase in particle content to 6%. It means that adding a small number of nano-SiO_2_ particles into the composites can promote the polymerization reaction. Then, the particles begin to hinder the DC as the particle content exceeds 6%, the DC of the composites decreases by 56.69%, and the blocking effect of the particles on the DC increases with particle contents.

#### 3.2.2. Fracture Morphology of Dental Resin Composites

**Figure 6:**
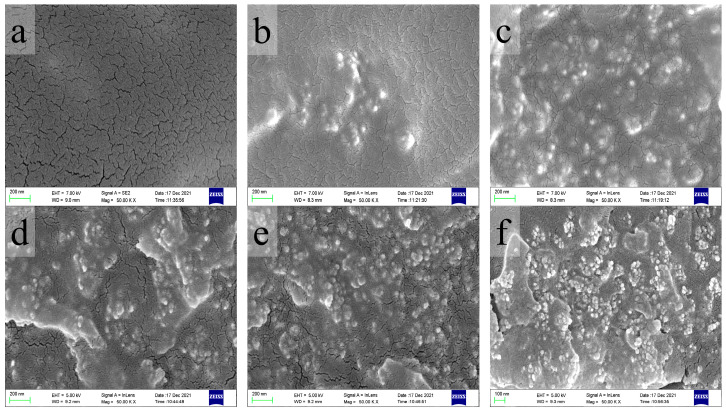
SEM images of the fractured sections after tensile tests for the composites with (**a**) 0; (**b**) 2 wt.%; (**c**) 4 wt.%; (**d**) 6 wt.%; (**e**) 8 wt.%; and (**f**) 10 wt.%.

Six representative SEM micrographs of one set of the fracture of samples after the tensile test with different particle contents are shown in Figure 6. As shown in Figure 6, the dental resin composites show a rougher fracture morphology with the rise of particle contents, which means more energy is required for the fracture. Additionally, in this work, the highest filling content of the nanoparticles is 10%, and the nanoparticles are not agglomerated at such low filling content (less than 10%) [9].

#### 3.2.3. The Interaction between Particles and Matrix

**Figure 7:**
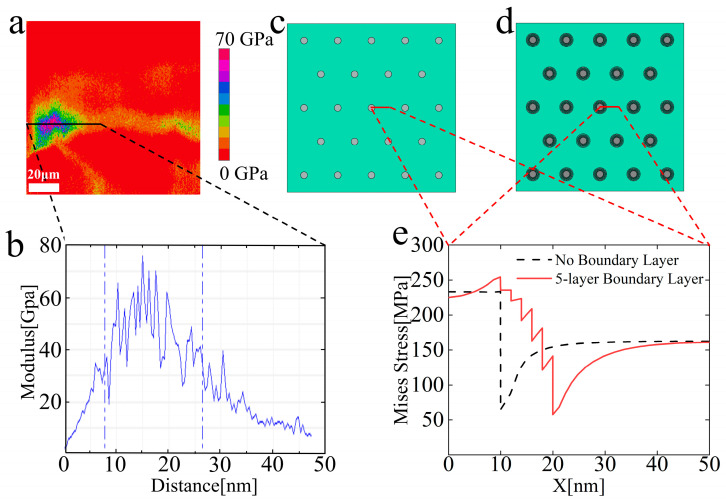
Results of the modulus mapping and finite element analysis. (**a**) A representative modulus map of the particle–matrix interfacial region of the composite; (**b**) Representative modulus profile of a scanning line shown in (**a**), showing the gradient variations of the modulus at the particle–matrix interfaces; (**c**) Finite element model without gradient boundary layers at the interfaces; (**d**) Finite element model with gradient boundary layers at the particle–matrix interfaces; (**e**) Distribution of the Mises stress at the interfacial region after loading.

The relevant studies of the filler–matrix interface are shown in Figure 7. A modulus distribution diagram of the composites measured by AFM is shown in Figure 7a. As shown in this picture, the modulus field around the particle would change; the modulus at the particle–matrix interface is not a cliff-type change, and there is an interfacial polymer layer of polymer composites. As Figure 7b shows, the modulus shows a trend similar to gradient change in this layer; it gradually decreases from the edge of the particle to the matrix and becomes stable at a certain distance from the particle. Therefore, a finite element simulation is performed by ABAQUS to explain the role of such a gradient boundary layer in the study of the strengthening mechanism of resin composites by particles. The finite element models in ABAQUS analyses are shown in Figure 7c (without the boundary layer) and Figure 7d (with the boundary layer). The thickness of the whole boundary layer was set to 10 nm, which is consistent with the thickness observed in Figure 7a. The boundary layer was divided into five layers with a decreasing modulus distribution from particle to resin matrix to simulate the modulus change of the boundary layer. The Mises stress distributions from the particle center to the resin matrix are shown in Figure 7e. Under the situation of no boundary layer, it can be seen that the stress at the filler–matrix interface drops from 230 MPa to 60 MPa almost instantaneously. However, the stress at the interface shows a trend of gradual decline when there is a boundary layer. In addition, the stress in the resin matrix is lower with the boundary layer. These phenomena are consistent with the results of a study on gradient fiber-reinforced composites [33].

## 4. Discussion

From the results in Figure 3, the resin composites showed better mechanical properties at higher test frequencies; it is caused by the low fluidity of polymer chains at high frequencies. Under constant stress, molecular chains within the polymer will rearrange themselves to resist the applied force and reduce the stress. There is not enough time for the chains to react adequately at high frequency. Increasing particle contents significantly improved the mechanical properties of the composites. These findings could be sued to choose appropriate resins for different patients in clinical trials. The obtained data in this work could also be applied to finite element simulation to calculate the shrinkage stress during clinical trials. On the one hand, the physical or chemical combination of inorganic particles and polymers produces action energy; the interface bond between particles and matrix can withstand the greater load, and the increasing of particle contents can contribute to the enlargement of the interface area, thus increasing the interface interaction. On the other hand, the existence of rigid inorganic particles will result in the stress concentration effect, which causes silver lines in the matrix and absorb the deformation work. The previous research found that the initiation and propagation of microcracks in the resin were limited by silica nanoparticles, which was clear evidence of toughness enhancement [34], and some investigations also found that a strong particle–matrix interface is due to crack deflection, particle pull-out, and plastic deformation, which resulted in the enhancement of the mechanical properties of resin composites [35]. The question of how the particles affect the deformation of the matrix polymer is still unclear, so a series of studies have been performed to explore it.

Essentially, the polymerization of composites is a chain polymerization of the monomer, and it is reflected by the DC. The final property of composites is significantly affected by the DC of the system. The higher the DC, the better the mechanical properties of dental resin composites. Our tests showed that the final DC of the composites first increased and then decreased with the addition of particles. The reason is that at the lower packing load, the packing particles are more evenly distributed, and multiple light scattering is carried out inside the composite, which improves the activation efficiency. The exceptionality of the nanoparticle structure endows them with strong photocatalytic performance, thus promoting the DC. However, the effect of the particles on improving light efficiency through light scattering is limited, and the promoting effect is not significant. At the higher packing load, the nanoparticles will agglomerate. When the size of the agglomerate reaches about 200 nm (it is close to half of the wavelength of visible light), the light scattering reaches the maximum, and the light-blocking effect is strong at this time, which reduces the irradiation efficiency. What is more, the higher contents of nanoparticles will reduce the mobility of the monomer and the free radicals, thus reducing the DC of the composites. The volume ratio of resin monomer is also reducing with the increase in nanoparticle contents, and the exothermic effect of polymerization decreases, thereby leading to the reduction in the DC. However, DC varied little with the particle contents, so DC was not the main factor in the mechanical properties of composites in this article.

As shown in Figure 6, there are some microcracks in the process of fracture. It is because the stress concentration occurs when the samples are subjected to the applied force, and it stimulates the microcracks in the resin matrix, which can absorb the energy from deformation. In addition, the existence of rigid inorganic particles will hinder the microcrack propagation of the resin matrix, avoid the appearance of macroscopic cracks, and then prevent the destructive cracking phenomenon. Moreover, the dental resin composites showed a rougher fracture morphology with an increase in particle contents. These phenomena are the same as in Wang’s study [34]. The more nanoparticles were added into the composites, the more microcracks were generated in the samples during the process of breaking, which made the roughness of the section after fracture increase. It is one of the reasons why the addition of particles could enhance the mechanical property of the composites.

The fundamental question of how the particles affect the deformation of the matrix polymer is still under active debate. For instance, some scholars thought that the filled matrix polymer held a larger effective deformation than in the unfilled state, invoking the concept of strain amplification or molecular overstraining [36,37,38,39]. Although the concept of strain amplification is widely employed by the polymer composite community, microscopic experiments regarding its existence are still inconclusive and controversial. Our results performed by AFM showed a gradient boundary layer. Sun and Wang [40] used small-angle neutron scattering to prove that the reason for the mechanical reinforcement of polymer composites was a theory that was akin to the hydrodynamic effect of nanoparticles rather than molecular overstraining. According to the classical hydrodynamic theory for dilute particle suspensions [41,42,43], the particles can distort the strain field around them, and such an effect propagates to the matrix. They proved that this theory could apply not only to fluids but also to the middle of the rubbery plateau, and our results further showed that this conclusion could be applied to the entire rubbery regime. Moreover, our work has also confirmed the reinforcement mechanism in the previous research. The strong particle–matrix interface is a gradient layer where the modulus gradually decreases from the edge of the particle to the matrix by AFM. In addition, the finite element simulation presented the effect of the boundary layer on stress in resin composites. The shear stress concentration on the filler–matrix interface can be greatly alleviated under the slow change of the modulus between the filler and the matrix, and the failure of the composite caused by interface debonding can be significantly postponed. The presence of the boundary layer can obviously alleviate the stress on the filler–matrix interface to enhance the mechanical properties of composites. The next work will focus on the effect of particle size on boundary layer thickness and enhancement effect.

## 5. Conclusions

The effects of filler content on the microscale and macroscale mechanical properties and the reinforcing mechanism of dental resin composites have been comparatively studied in a systematic manner. With the particle content increasing from 0 to 10 wt.%, the nanoindentation modulus and hardness of the composites increase by 36.27% and 40.90%, respectively. The macroscale tensile modulus and ultimate tensile strength increase from 2.47 GPa and 36.22 MPa to 3.17 GPa and 51.75 MPa, respectively. A series of characterizations have been performed to explore the strengthening mechanism of the resin composites. It is found that the variation of the double-bond conversion of the composites is too slight to be the major factor for the mechanical reinforcement. Morphology characterizations of the fractured samples indicate that the roughness of the sections after fracture gradually increases with the addition of the nano-silica particles. Moreover, a gradient boundary layer with decreasing elastic modulus was found for the particle–matrix interfaces. Finite element analysis demonstrates the advantage of this gradient interfacial layer in alleviating the stress concentrations and thus facilitating the mechanical reinforcement for the composites. The findings in this work not only provide comparative results of the mechanical properties of dental composites at different scales but also open up a potential new insight into the reinforcing mechanisms for general polymer nanocomposites.

## Data Availability

Not applicable.
